# Near-Infrared Fluorescence Imaging in Preclinical Models of Glioblastoma

**DOI:** 10.3390/jimaging9100212

**Published:** 2023-10-06

**Authors:** Monserrat Llaguno-Munive, Wilberto Villalba-Abascal, Alejandro Avilés-Salas, Patricia Garcia-Lopez

**Affiliations:** 1 Laboratorio de Fármaco-Oncología, Subdirección de Investigación Básica, Instituto Nacional de Cancerología, Mexico City 14080, Mexico; muniv1250@hotmail.com (M.L.-M.); wilbertovillalba@gmail.com (W.V.-A.); 2 Laboratorio de Física Médica, Subdirección de Investigación Básica, Instituto Nacional de Cancerología, Mexico City 14080, Mexico; 3 Departamento de Patología, Instituto Nacional de Cancerología, Mexico City 14080, Mexico; alejandroaviles2001@yahoo.com

**Keywords:** near-infrared fluorescence, glioblastoma, optical imaging

## Abstract

Cancer is a public health problem requiring ongoing research to improve current treatments and discover novel therapies. More accurate imaging would facilitate such research. Near-infrared fluorescence has been developed as a non-invasive imaging technique capable of visualizing and measuring biological processes at the molecular level in living subjects. In this work, we evaluate the tumor activity in two preclinical glioblastoma models by using fluorochrome (IRDye 800CW) coupled to different molecules: tripeptide Arg-Gly-Asp (RGD), 2-amino-2-deoxy-D-glucose (2-DG), and polyethylene glycol (PEG). These molecules interact with pathological conditions of tumors, including their overexpression of αvβ3 integrins (RGD), elevated glucose uptake (2-DG), and enhanced permeability and retention effect (PEG). IRDye 800CW RGD gave the best in vivo fluorescence signal from the tumor area, which contrasted well with the low fluorescence intensity of healthy tissue. In the ex vivo imaging (dissected tumor), the accumulation of IRDye 800CW RGD could be appreciated at the tumor site. Glioblastoma tumors were presently detected with specificity and sensitivity by utilizing IRDye 800CW RGD, a near-infrared fluorophore combined with a marker of αvβ3 integrin expression. Further research is needed on its capacity to monitor tumor growth in glioblastoma after chemotherapy.

## 1. Introduction

Glioblastoma is a lethal cancer of the central nervous system that continues to gravely affect human health despite medical and technological advances. After the standard treatment, which consists of surgical resection, radiotherapy, and adjuvant temozolomide chemotherapy, the mean overall survival time is from 12 to 14 months and patients die within 2 years of their diagnosis [1,2].

The challenges of glioblastoma prevention, detection, and treatment have stimulated the development of new diagnostic drugs and technologies [3,4,5]. During the last few decades, different non-invasive imaging techniques have been developed for monitoring tumor growth in preclinical and clinical settings. Imaging strategies, including computerized tomography (CT), positron emission tomography (PET), and magnetic resonance (MRI), allow for the detection and measurement of cellular and molecular processes in live animals [3,4,6]. Radionuclide imaging has advantages, such as good sensitivity, but is often costly. Moreover, it provides low spatial resolution and requires personnel trained in radiation.

Hence, optical (fluorescence and bioluminescence) imaging has emerged as an attractive non-invasive modality. Fluorescence imaging in the biomedical field has distinct advantages in terms of sensitivity, selectivity, response time, and safety [7]. Unlike nuclear imaging techniques, optical imaging does not involve any ionizing radiation. In fluorescence imaging, the light emission stimulated by a laser or other external light source poses no risk. In addition, there are a wide range of suitable fluorophores available, and these switch from a fluorescently inactive to an emitting state in the presence of certain biological events [8].

One fluorescence imaging technique, near-infrared fluorescence, is generally preferred for in vivo imaging. Due to its lower absorption by hemoglobin and water, it penetrates deeper into tissue than visible-range light. Furthermore, less autofluorescence exists in surrounding tissues, resulting in low background signal intensity [9,10]. An excellent signal-to-background ratio is desirable in any molecular imaging technique within its broad wavelength spectrum, which is from 650 to 900 nm for near-infrared fluorescence.

Numerous compounds with potential for targeted therapy have been employed for fluorescence imaging. These include small molecules, peptides, proteins, and antibody-based ligands [11]. The main strategy for developing specific markers is to take advantage of all the ligand-receptor, enzyme-substrate, and antibody–antigen interactions that may be useful for practical approaches to imaging [11,12].

Techniques based on near-infrared fluorescence have been evaluated for their capacity to identify tumors in animal models and in the intraoperative protocol in patients. Although there are various near-infrared fluorescence dyes, only a few have characteristics suitable for capturing images in heterogeneous tissues in vivo. During the last decade, the search for an optimized fluorescent probe has been a promising area of research [13,14,15]. Newton et al. carried out intraoperative near-infrared imaging with an FDA-approved near-infrared fluorescence optical contrast agent to identify mammary tumors in dogs [16]. Several studies on patients have shown that near-infrared fluorescence can be utilized to identify tumors and thus guide surgery [17,18,19]. Three non-specific fluorophores have been approved for clinical use by the United States Food and Drug Administration (FDA) and the European Medicines Agency (EMA): indocyanine green (ICG), methylene blue (MB), and 5-aminolevulinic acid (5-ALA). However, only 5-ALA has been beneficial for glioblastoma patients [9].

Both positron emission topography (PET) and fluorescence produce molecular images based on cell labeling. Labeling agents target the distinctive characteristics of tumor cells resulting from alterations in normal physiological processes. For example, the accelerated rate of angiogenesis of tumor cells involves an elevated expression of αvβ3 integrins, which are heterodimeric glycoproteins on the cell surface in cell-to-cell and cell-to-matrix interactions. The ability to non-invasively image αvβ3 integrin expression in living subjects would represent a novel approach to the diagnosis of tumors and metastases and would likely provide new insights into angiogenesis in tumors [20]. One substance that binds to integrin receptors, including αvβ3 integrins, is the tripeptide Arg-Gly-Asp (RGD). This recognition motif is contained in IRDye 800CW RGD, a BrightSite™ near-infrared fluorescence-labeled RGD imaging agent.

Another alteration common to many tumor microenvironments is the enhanced permeability and retention (EPR) effect of discontinuous vascular endothelium [21]. It can be explored with polyethylene glycol (PEG), a soluble synthetic polymer that has been conjugated with a fluorophore to form the IRDye 800CW PEG contrast agent [22].

Additionally, malignant tumors are characterized by a high demand for glucose (even under aerobic conditions), which involves glucose uptake through glucose transporters (GLUT). 2-Amino-2-deoxy-D-glucose (2-DG), a glucose analog that utilizes GLUT transporters to enter cells, is not metabolized further after its phosphorylation, effectively being trapped within the cell. IRDye 800CW 2-DG has proven to bind to the cells of multiple tumor types in both in vitro assays and in vivo animal models [23].

Hence, the administration of selective markers permits the differentiation of images of tumor cells from healthy cells. The markers take advantage of the distinctive characteristics of tumor cells. The combination of a marker with imaging technology, such as near-infrared fluorescence imaging, holds great promise for monitoring cancer progression and helping to explore cancer mechanisms. The aim of the current contribution was to use near-infrared fluorescence imaging to non-invasively monitor tumor growth in two preclinical models of glioblastoma. Accordingly, fluorochrome (IRDye 800CW) was coupled to RGD, PEG, and 2-DG to evaluate tumor activity. This type of imaging tool has the potential of improving the diagnosis of tumors and aiding research into cancer therapy by providing insights into tumors at a molecular level.

## 2. Materials and Methods

### 2.1. Reagents

The three imaging agents herein tested, IRDye 800CW conjugated with RGD, 2-DG, or PEG, were supplied by LI-COR Bioscience (Lincoln, NB, USA). Dulbecco’s modified Eagle’s medium (DMEM), Roswell Park Memorial Institute medium (RPMI), fetal bovine serum (FBS), and ethylenediaminetetraacetic acid (EDTA) were purchased from Gibco (Grand Island, NY, USA).

### 2.2. Cell Cultures

Rat C6 glioma cells and human U87-MG glioblastoma cells were obtained from the American Type Culture Collection (ATCC, Manassas, VA, USA) and maintained at 37 °C in a 5% CO_2_ humidified atmosphere in DMEM supplemented with 5% FBS, and RPMI supplemented with 10% FBS, respectively.

### 2.3. Animals

Male nu/nu mice (20–25 g) and male Wistar rats (200–250 g) were supplied by the Universidad Autónoma Metropolitana (UAM) and the Facultad de Medicina of the Universidad Autónoma de México (UNAM), respectively (both institutions in Mexico City). All animals were provided food and water ad libitum. They were kept in pathogen-free conditions on a 12/12 h light/dark cycle at 27 °C with adequate humidity. The experiments were performed in accordance with the rules for the care and use of experimental animals approved by the Ethics Committee of the National Cancer Institute of Mexico (010-17)-(IBICB601-10) and the “Technical specifications for the production, care and use of laboratory animals” published by the Secretary of Agriculture in Mexico (SAGARPA, NOM-062-ZOO-1999) [24].

### 2.4. Tumor Xenografts

Nude mice were subcutaneously (s.c.) inoculated with 1 × 106 C6 cells or 3 × 106 U87-MG cells in the right flank. Subsequently, the tumor of the mice was periodically measured with a caliper, and tumor volume was determined with the following formula V = D × d^2^ × (π/6) (D, large diameter; d, short diameter). Once tumors had reached approximately 150–200 mm^3^, optical images were acquired with the Odyssey Infrared Imaging System (LI-COR Bioscience, Lincoln, NB, USA).

### 2.5. C6 Cell Orthotopic Implantation in Wistar Rats

C6 tumor cells were implanted in Wistar rats as described by Llaguno et al. [25]. Briefly, each animal was anesthetized with tiletamine hydrochloride (10 mg/kg s.c.) and acepromazine maleate (0.4 mg/kg s.c.) then placed in a stereotactic device for surgery. With an infusion pump, 7.5 × 105 C6 glioma cells were orthotopically implanted intracranially in Wistar rats at the coordinates of 2.0 mm right from bregma and 6 mm deep with a rate of 0.5 μL/min.

### 2.6. U87-MG Cell Orthotopic Implantation in Nude Mice

U87-MG cells were inoculated intracranially into nu/nu mice by means of stereotaxic surgery with a 27G gauge needle. While mice were maintained under anesthetic conditions with an isoflurane/oxygen system, an incision was made 2 mm to the right and 1 mm posterior to the Bregma, and 7.5 × 105 U87-MG glioma cells were orthotopically deposited 2.5 mm deep at a rate of 0.5 μL/min with an infusion pump.

### 2.7. In Vivo and Ex Vivo Imaging

The fluorescent markers (1 nmol) were injected intravenously into animals, and the optical images were acquired at 0, 2, 18, 24, 48, and 72 h post-injection. The animals were anesthetized with 2% isoflurane throughout the imaging procedure and the images were captured with the Odyssey Infrared Imaging System (LI-COR Bioscience, Lincoln, NB, USA).

**Figure 1:**
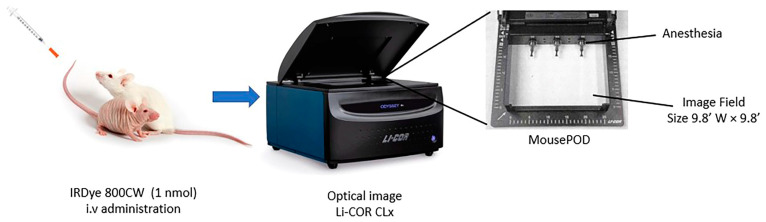
Odyssey Infrared Imaging System (Li-COR CLx) used for acquiring images with different IRDye-800W probes.

We used the 800 nm wavelength (Channel Laser Source) with an excitation filter of 785 nm and an emission filter of 820 nm with laser power of 50 nW. The Image Field Size was 9.8 inch W × 9.8 inch D (Figure 1). The settings are shown in Table 1. Focus was defined as the sharpness and clarity of the image, and metrics such as peak signal-to-noise ratio (PSNR) were used to evaluate image quality and quantify. PSNR measures the ratio of the peak signal power to the noise power.

The animals were sacrificed 48 h post-administration of fluorescent markers and the brains were dissected for optical images.

For each analysis of different probes, the images have been normalized to the same fluorescent scale (LUT) to optimize their analysis. This normalization step was implemented to facilitate visual comparisons across all images. However, LUT was not required for quantification.

Regions of interest were selected and quantified for total pixel values, and the background fluorescence was subtracted from the signal intensities. The images were analyzed with Image Studio Lite (Version 5.2).

### 2.8. Histological Staining

For histological analysis, animals were euthanized three weeks post-inoculation and brains were harvested and fixed in 10% formaldehyde. They were then embedded into paraffin blocks, which were sliced on a microtome and stained with H&E.

### 2.9. Statistical Analysis

The values are expressed as the mean (n = 3) ± SD. The statistical analyses were performed on GraphPad Prism software version 8.0.1, comparing the data between groups with one-way analysis of variance (ANOVA). Significant differences were considered at *p* < 0.05.

## 3. Results

### 3.1. IRDye 800CW RGD

**Figure 2:**
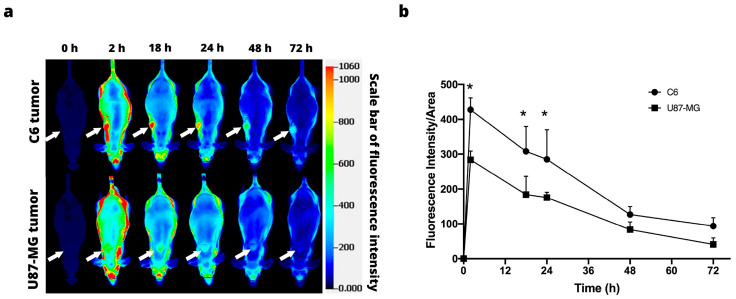
In vivo fluorescence imaging subsequent to intravenous injection of IRDye 800CW RGD. (**a**) Fluorescence imaging of C6 (above) and U87-MG tumors (below). The position of the tumor is indicated by the white arrows. (**b**) Fluorescence intensity as a function of the time elapsed after the subcutaneous administration of C6 and U87-MG xenografts. Values represent the mean (n = 3) ± SD. * significant difference (*p* < 0.05) between C6 tumor with U87-MG tumor.

IRDye 800CW RGD was injected intravenously into animals, and the intratumoral accumulation of IRDye 800CW RGD was examined in vivo in the U87-MG and C6 tumors at 0, 2, 18, 24, 48, and 72 h post-injection, finding a difference between the two. Whereas the accumulation of the IRDye 800CW RGD continued in the C6 tumors for up to 72 h, it diminished in the U87-MG tumors after 48 h. In both cases, sufficient contrast of fluorescence existed between the tumor and the rest of the body, thus furnishing a clearly delimited tumor image (Figure 2a).

The fluorescence signal intensity was quantified during 72 h post-injection of the fluorescence marker, based on the establishment of a region of interest in the tumor. In both cell lines, the signal intensity was at its peak at 2 h post-injection, followed by a gradual decrease over time (Figure 2b).

### 3.2. IRDye 800CW PEG

**Figure 3:**
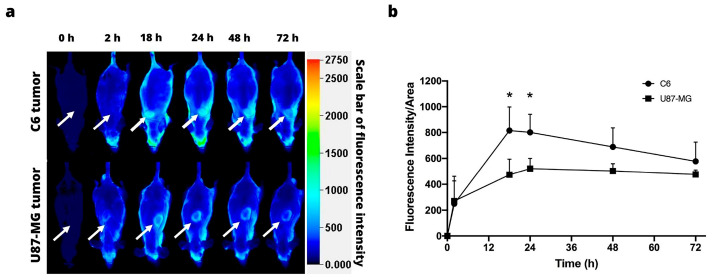
In vivo fluorescence imaging subsequent to intravenous injection of IRDye 800CW PEG. (**a**) Fluorescence imaging of the C6 (above) and U87-MG tumors (below). The position of the tumor is indicated by the white arrows. (**b**) Fluorescence intensity as a function of the time elapsed after the subcutaneous administration of C6 and U87-MG xenografts. Values represent the mean (n = 3) ± SD. * significant difference (*p* < 0.05) between C6 tumor with U87-MG tumor.

The tumor retention of the IRDye 800CW PEG is shown in Figure 3a. The macromolecule was visible at 18 h post-injection in the C6 xenograft model of glioblastoma, but only visible in the periphery of the tumor at 18 h post-injection in the U87-MG xenograft model (Figure 3a). In both cell lines, the signal intensity peaked at 18 h post-injection, and gradually decreased for the next 54 h (Figure 3b).

### 3.3. IRDye 800CW 2-DG

**Figure 4:**
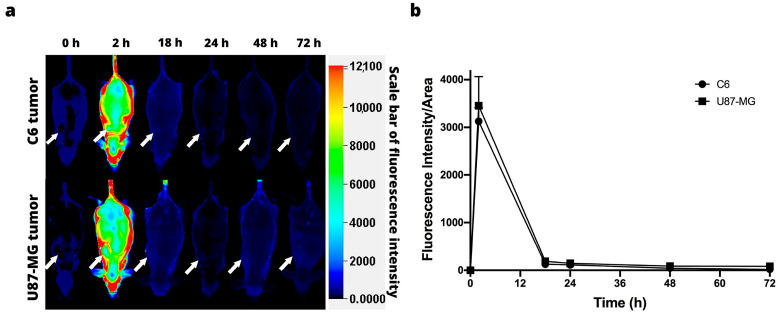
In vivo fluorescence imaging subsequent to intravenous injection of IRDye 800CW 2-DG. (**a**) Fluorescence imaging of nude mice with a subcutaneous C6 (above) or U87-MG tumor (below). The position of the tumor is indicated by the white arrows. (**b**) Fluorescence intensity as a function of the time elapsed after the subcutaneous administration of C6 and U87-MG xenografts. Values represent the mean (n = 3) ± SD.

Figure 4a illustrates the images acquired with the Odyssey Infrared Imaging System (LI-COR) at 0, 2, 18, 24, 48, and 72 h post-injection of IRDye 800CW-2-DG in the animals of the C6 and U87-MG xenograft models. Since the fluorescence signal was intense at 2 h post-injection, its distribution being uniform throughout the bodies of the mice, the tumor area could not be differentiated for either cell line (Figure 4b).

### 3.4. Orthotopic Models

**Figure 5:**
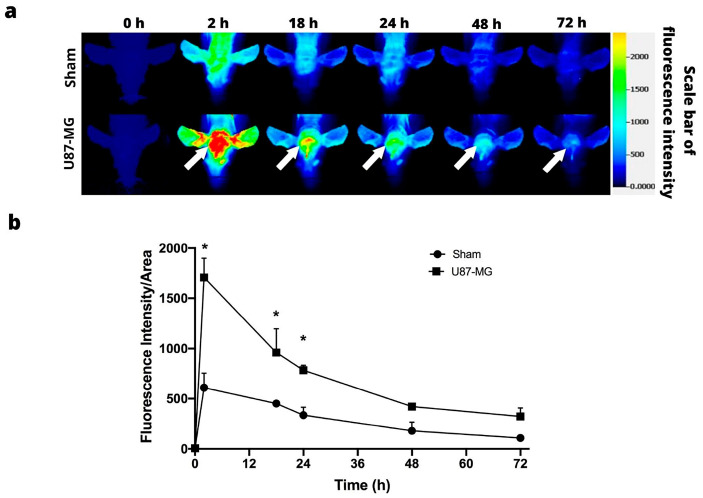
In vivo fluorescence imaging of IRDye 800CW RGD uptake by U87-MG intracranial glioma cells. (**a**) Fluorescence imaging of nude mice with an orthotopic U87-MG tumor. The position of the tumor is indicated by the white arrows. (**b**) Fluorescence intensity as a function of the time elapsed after the U87-MG xenografts were administered or the sham operation performed. Values represent the mean (n = 3) ± SD. * A significant difference (*p* < 0.05) between Sham group with implanting of glioma cells U87-MG.

In the U87-MG orthotopic inoculation, in both the sham and tumor groups, near-infrared fluorescence images showed high fluorescence intensity at 2 h. At 18 h, the probe had become more concentrated in the tumor area. In contrast, in the sham group, the IRDye 800CW RGD was distributed throughout the brain. In both groups, the fluorescence intensity decreased over time (Figure 5a). There was a very notable difference in signal intensity between the mice inoculated with tumor cells and the sham animals (Figure 5b).

**Figure 6:**
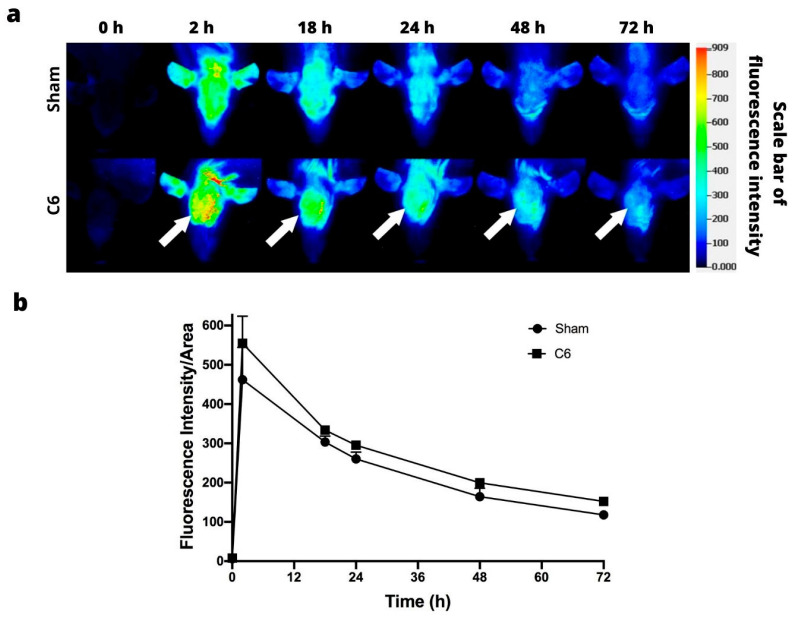
In vivo fluorescence imaging of IRDye 800CW RGD uptake by C6 intracranial glioma cells. (**a**) Fluorescence imaging of rats with an orthotopic C6 tumor. The position of the tumor is indicated by the white arrows. (**b**) Fluorescence intensity as a function of the time elapsed after the C6 tumors were administered or the sham operation performed. Values represent the mean (n = 3) ± SD.

Unlike the orthotopic model in athymic mice, in Wistar rats, it was impossible to adequately differentiate the tumor tissue from the healthy tissue (Figure 6a). Although both species of animals were shaven, in the rats, the tumor could not be differentiated from surrounding tissue, and no difference was observed in the fluorescence intensity between the sham and inoculation groups (Figure 6b).

#### Ex Vivo Imaging and Histology Analysis

**Figure 7:**
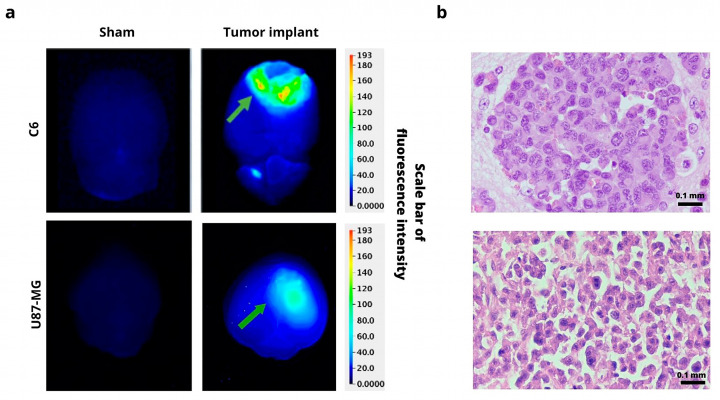
(**a**) Ex vivo fluorescence imaging of IRDye 800CW RGD in the brain of the sham-operated group (without any tumor, **left**) and of animals with a tumor implanted (**right**) at 48 h post-injection of 800CW RGD. (**b**) H&E staining of C6 and U87-MG tumor tissue. Green arrows indicate tumor location. Scale bar = 0.1 mm. 40×.

In order to confirm the presence of a tumor, animals were sacrificed at 48 h post-injection of 800CW RGD. A difference was found in the fluorescence intensity of the ex vivo images of the normal brain tissue (the sham group) and the animals with a tumor implanted, both in the C6 and U87-MG models. The fluorescence of 800CW RGD was observed in the tumor groups but not in the sham animals (Figure 7a). The fluorescence signal is generated only at the site where the marker accumulates (due to the binding of IRDye 800CW RGD to αvβ3 integrins). The H&E stain provides a visualization of the high degree of cellular density and pleomorphism that is typical of glioblastoma (Figure 7b). These stains correspond to the fluorescence imaging results of the same tumor tissue with IRDye 800CW RGD.

## 4. Discussion

One of the fundamental limitations in cancer research and therapy is the ability to track tumor growth and thus determine the efficacy of new cancer treatments. Consequently, the search for new diagnostic techniques is key to achieving new advances. In this sense, in vivo imaging techniques are essential in biomedicine and cell biology. Regarding medicine, they are advantages for drug discovery and development as well as for the clinical assessment of central nervous system diseases. These techniques allow for the localization of tumors, the visualization of the expression and activity of specific molecules, and the monitoring of biological processes that influence tumor behavior and/or response to therapy [26,27,28]. Near-infrared dye penetrates deeper into tissue and offers low scatter and background fluorescence in healthy tissue compared with other near-infrared fluorophores [27].

The different commercial probes herein evaluated for their capacity to accumulate in glioblastoma tumor models (orthotopic and xenograft) were 2-DG, RGD, and PEG. They were coupled to a fluorochrome that emits near-infrared wavelengths, which have deeper penetration into tissue and greater sensitivity than visible-range light.

The three imaging agents were evaluated at different times to choose the best optimal time. Choosing the optimal imaging time point is crucial for optimizing the upcoming studies. It is essential to identify when the signal-to-noise ratio is at its highest. It is crucial to determine the time that necessary to clear the probe adequately on the non-specific sides to avoid the fluorescence throughout the animal being too intense to image accurately. Through this analysis, we visualize a gradual reduction in signal intensities over time due to the cleared probe on the non-specific sides. It is generally preferred to analyze a significantly higher signal than the background.

Each probe has a different uptake depending on its biological affinity for the receptors expressed in the tumor cells. This information can guide the design of more targeted experiments and help optimize the use of resources and time. To validate the specificity of the tracer, we included negative controls with non-specific agents, such as saline solution basal image, and positive controls to the cell or target-of-interest could confirm specificity and demonstrate the tumor localization. If the receptor is higher in tumors, the tracers bind more than in the case of the normal cells.

Since the RGD peptide binds selectively to αvβ3 integrins, it has been coupled with a fluorescent marker in various studies to evaluate tumor growth in animal models [29,30]. Due to the fact that integrins, which are a family of heterodimeric cell surface receptors expressed on a wide variety of tumors, are responsible for regulating crucial cellular functions such as angiogenesis, adhesion, migration, and invasion [31], in this study, the intratumoral accumulation of IRDye 800CW RGD could be detected and adequately quantified in both cancer cell lines, and there was greater signal intensity for the C6 line. While this difference may owe itself to a distinct expression of αvβ3 integrins in the cell lines, contradictory reports are found on such expression. Hence, one of the future objectives will be to determine the level of expression of αvβ3 integrins of C6 and U87-MG cells. Considering that an association has been reported between a high expression of αvβ3 integrins and a poor prognosis in glioblastoma [32], these integrins may be an important candidate for the in vivo analysis of tumor progression in animal models.

On the other hand, the enhanced permeability and retention (EPR) effect has been accepted as one of the pathophysiological characteristics of solid tumors. Since larger molecules reportedly tend to accumulate in tumors [21], a pegylated dye (24–60 kDa) was utilized to assess the retention of labeled macromolecules. In this work, the IRDye 800CW PEG was only visible in the periphery of the tumor at 18 h post-injection in the U87-MG xenograft model, in comparison with the C6 xenograft, where the probe was visible after 18 h hours post-inoculation. In contrast with the IRDye 800CW RGD probe, the signal intensity decreased more slowly with the PEG probe. PEG is a macromolecule retained in discontinuous vessels, making its elimination more difficult. With near-infrared fluorescence agents that target either αvβ3 integrins or the EPR effect, Keereweer et al. could detect tumors in an orthotopic mouse model of oral cancer [22]. However, there are some limitations to the application of IRDye 800CW PEG because it is not a tumor-specific target.

Another essential characteristic that distinguishes cancer cells from healthy cells is the elevated glucose metabolism. A number of molecular imaging techniques utilize this metabolic difference to monitor tumor progression with a glucose analog, 2-amino-2-deoxy-D-glucose (2-DG). Although there are studies in which the accumulation of 2-deoxy-2-(18F) fluoro-D- glucose (18F-FDG) within the tumor is evaluated with microPET in various animal models of cancer, it is a more expensive technique and requires personnel trained in radiation. Thus, the use of 2-DG labeled with a fluorochrome has often been preferred. IRDye 800CW 2-DG was presently examined as a possible molecular probe in a glioblastoma model.

Various near-infrared fluorescence 2-deoxyglucose analogs have been successfully synthesized for non-invasive optical tumor imaging. In this work, we evaluated the tumor retention of the IRDye 800CW 2-DG at different times. In the case of the subcutaneous glioblastoma model, no difference in fluorescence intensity was observed between the tumor and healthy tissue. Other studies with 2-DG probes have shown an accumulation of 2-DG in the tumor. For instance, Cy5.5 has been shown to have a higher uptake in subcutaneous U87-MG glioblastoma tumors than in normal tissue [33]. On the other hand, the tumor uptake was greater for FDG than Cy5.5 2-DG, perhaps because of the unspecific binding of Cy5.5. Kovar et al. reported a specific distribution of IRDye 800CW 2-DG within tumors in athymic nude mice bearing prostate, epidermoid, and colorectal tumors [23]. Due to the accelerated rate of glucose accumulation in the tumor, the time points for making measurements in this study may have been too far apart, suggesting the need to evaluate several times points between 2 and 18 h post-injection.

Several studies have been reported the use of NIRF probes to evaluated the growth tumor in an orthotopic model of glioma. For an orthotopic inoculation, physiological and biological characteristics were considered for the probe, such as the depth of injection, the blood–brain barrier, vascularization, and the elevated metabolic activity of glucose. It was decided to discard the evaluation of the 2-DG probe given that the high glucose metabolism in the brain impeded the delimitation of the tumor from the surrounding tissue. PEG was also discarded because of its large size, which did not allow it to cross the blood–brain barrier. Hence, RGD was selected as the most appropriate marker for this study due to the overexpression of αvβ3 in glioma cells. In the U87-MG orthotopic model, near-infrared fluorescence images showed a difference in signal intensity between the mice inoculated with tumor cells and the sham group. For the orthotopic model of the C6 cell line, Wistar rats were the animals of choice because they would not produce rejection of the implant, being the same species as the animals of tumor origin. However, in Wistar rats, it was impossible to adequately differentiate tumor tissue from healthy tissue. Perhaps the thicker bone density in the rat versus the mouse skull impedes the adequate capture of fluorescence. Additionally, the tumor was located deeper under the skin in the rats than in the mice. However, in order to confirm the presence of a tumor, animals were sacrificed at 48 h post-injection of 800CW RGD. A difference was found in the fluorescence intensity of the ex vivo images of the brains of the sham group compared to images of the brains of rats implanted orthotopically with tumor cells (C6 or U87-MG). The fluorescence of 800CW RGD was observed in the tumor groups but not in the sham animals. Therefore, in rats, only ex vivo brains with C6 tumors are available to carry out trials. The fluorescence signal is generated only at the site where the marker accumulates (due to the binding of IRDye 800CW RGD to αvβ3 integrins), and the fluorescence imaging of tumors corresponds with histological imaging where the H&E stain showed a high degree of cellular density that is typical in glioblastoma tumors.

The use of fluorescence techniques has already been important in preclinical and medical images due to a non-invasive imaging technique that can help visualize biological processes. Using different techniques could allow us to acquire more information about the biological process to be studied, and combining several techniques could enrich the discussion and expand the horizons of the research. The use of fluorescence lifetime imaging microscopy (FLIM) has been employed in biomedical applications because FLIM can evaluate different processes in cells, tissues, disease progression, and drug efficacy [34,35]. The additional information provided by several imaging techniques could contribute to more accurate differentiation between various cellular components or molecular interactions.

## 5. Conclusions

Near-infrared fluorescence is a good non-invasive imaging tool. It was presently combined with three different markers (RGD, 2-DG, and PEG) to test the capacity of each to monitor the growth of two models of glioblastoma tumors (C6 and U87-MG). Better results were found with IRDye 800CW RGD than IRDye 800CW 2-DG or IRDye 800CW PEG. The markers interact with αvβ3 integrins, glucose, and the enhanced permeability and retention effects, respectively. There was substantially more IRDye 800CW RGD accumulated in tumor versus healthy tissue, which is due to the high expression of αvβ3 integrins in the former. Hence, this fluorescence dye could possibly be used to interact with tumors to afford greater accuracy in evaluations of preclinical studies and in diagnoses after novel cancer therapy. Although further research is necessary, IRDye 800CW RGD holds promise as a means of providing information beneficial for personalized medicine.

## Figures and Tables

**Table 1:** Image acquisition settings of Odyssey Infrared Imaging System (LI-COR Bioscience, Lincoln, NB, USA).

Parameters	Xenograft Model	Orthotopic Model
Focus (mm)	4.0	0.5
Resolution (µm)	337	337
Quality	Medium	Medium
Depth of field (mm)	4.0	4.0
Penetration depth (mm)	4.0	4.0
Wavelength (nm)	800	800

## Data Availability

Data is contained within the article.

## References

[1] Thust S.C., van den Bent M.J., Smits M. (2018). Pseudoprogression of brain tumors. J. Magn. Reson. Imaging.

[2] Chinot O.L., Wick W., Mason W., Henriksson R., Saran F., Nishikawa R., Carpentier A.F., Hoang-Xuan K., Kavan P., Cernea D. (2014). Bevacizumab plus radiotherapy-temozolomide for newly diagnosed glioblastoma. N. Engl. J. Med..

[3] Qin D., Yang G., Jing H., Tan Y., Zhao B., Zhang H. (2022). Tumor Progression and Treatment-Related Changes: Radiological Diagnosis Challenges for the Evaluation of Post Treated Glioma. Cancers.

[4] Higgins L.J., Pomper M.G. (2011). The evolution of imaging in cancer: Current state and future challenges. Semin. Oncol..

[5] Rong L., Li N., Zhang Z. (2022). Emerging therapies for glioblastoma: Current state and future directions. J. Exp. Clin. Cancer Res..

[6] Wang Z.J., Chang T.-T.A., Slauter R., Faqi A.S. (2013). Chapter 32—Use of Imaging for Preclinical Evaluation. A Comprehensive Guide to Toxicology in Preclinical Drug Development.

[7] Orrit M., Berberan-Santos M.N. (2008). Fluorescence as the Choice Method for Single-Molecule Detection. Fluorescence of Supermolecules, Polymers, and Nanosystems.

[8] Keereweer S., Van Driel P.B., Snoeks T.J., Kerrebijn J.D., Baatenburg de Jong R.J., Vahrmeijer A.L., Sterenborg H.J., Lowik C.W. (2013). Optical image-guided cancer surgery: Challenges and limitations. Clin. Cancer Res..

[9] Neijenhuis L.K.A., de Myunck L., Bijlstra O.D., Kuppen P.J.K., Hilling D.E., Borm F.J., Cohen D., Mieog J.S.D., Steup W.H., Braun J. (2022). Near-Infrared Fluorescence Tumor-Targeted Imaging in Lung Cancer: A Systematic Review. Life.

[10] Kosaka N., Ogawa M., Choyke P.L., Kobayashi H. (2009). Clinical implications of near-infrared fluorescence imaging in cancer. Future Oncol..

[11] Hilderbrand S.A., Weissleder R. (2010). Near-infrared fluorescence: Application to in vivo molecular imaging. Curr. Opin. Chem. Biol..

[12] Lwin T.M., Turner M.A., Amirfakhri S., Nishino H., Hoffman R.M., Bouvet M. (2022). Fluorescence Molecular Targeting of Colon Cancer to Visualize the Invisible. Cells.

[13] Yang R.Q., Chen M., Zhang Q., Gao Y.Y., Lou K.L., Lin T.T., Huang W.H., Zeng Y.Z., Zhang Y.Q., Dang Y.Y. (2022). Development and Preclinical Evaluation of a Near-Infrared Fluorescence Probe Based on Tailored Hepatitis B Core Particles for Imaging-Guided Surgery in Breast Cancer. Int. J. Nanomed..

[14] Hernot S., van Manen L., Debie P., Mieog J.S.D., Vahrmeijer A.L. (2019). Latest developments in molecular tracers for fluorescence image-guided cancer surgery. Lancet Oncol..

[15] Luo X., Li J., Zhao J., Gu L., Qian X., Yang Y. (2019). A general approach to the design of high-performance near-infrared (NIR) D-π-A type fluorescent dyes. Chin. Chem. Lett..

[16] Newton A., Predina J., Mison M., Runge J., Bradley C., Stefanovski D., Singhal S., Holt D. (2020). Intraoperative near-infrared imaging can identify canine mammary tumors, a spontaneously occurring, large animal model of human breast cancer. PLoS ONE.

[17] Tipirneni K.E., Warram J.M., Moore L.S., Prince A.C., de Boer E., Jani A.H., Wapnir I.L., Liao J.C., Bouvet M., Behnke N.K. (2017). Oncologic Procedures Amenable to Fluorescence-guided Surgery. Ann. Surg..

[18] Zhou Q., van den Berg N.S., Rosenthal E.L., Iv M., Zhang M., Vega Leonel J.C.M., Walters S., Nishio N., Granucci M., Raymundo R. (2021). EGFR-targeted intraoperative fluorescence imaging detects high-grade glioma with panitumumab-IRDye800 in a phase 1 clinical trial. Theranostics.

[19] Gilmore D.M., Khullar O.V., Jaklitsch M.T., Chirieac L.R., Frangioni J.V., Colson Y.L. (2013). Identification of metastatic nodal disease in a phase 1 dose-escalation trial of intraoperative sentinel lymph node mapping in non-small cell lung cancer using near-infrared imaging. J. Thorac. Cardiovasc. Surg..

[20] Jin H., Varner J. (2004). Integrins: Roles in cancer development and as treatment targets. Br. J. Cancer.

[21] Wu J. (2021). The Enhanced Permeability and Retention (EPR) Effect: The Significance of the Concept and Methods to Enhance Its Application. J. Pers. Med..

[22] Keereweer S., Mol I.M., Kerrebijn J.D., Van Driel P.B., Xie B., Baatenburg de Jong R.J., Vahrmeijer A.L., Lowik C.W. (2012). Targeting integrins and enhanced permeability and retention (EPR) effect for optical imaging of oral cancer. J. Surg. Oncol..

[23] Kovar J.L., Volcheck W., Sevick-Muraca E., Simpson M.A., Olive D.M. (2009). Characterization and performance of a near-infrared 2-deoxyglucose optical imaging agent for mouse cancer models. Anal. Biochem..

[24] [NOM-062- ZOO-1999]. Diario Oficial de la Federación, México. 15. Organización Mundial de Sanidad Animal. https://www.gob.mx/cms/uploads/attachment/file/203498/NOM-062-ZOO-1999_220801.pdf.

[25] Llaguno-Munive M., Romero-Pina M., Serrano-Bello J., Medina L.A., Uribe-Uribe N., Salazar A.M., Rodriguez-Dorantes M., Garcia-Lopez P. (2018). Mifepristone Overcomes Tumor Resistance to Temozolomide Associated with DNA Damage Repair and Apoptosis in an Orthotopic Model of Glioblastoma. Cancers.

[26] Weissleder R. (2006). Molecular imaging in cancer. Science.

[27] Zhang H., Uselman R.R., Yee D. (2011). Exogenous near-infrared fluorophores and their applications in cancer diagnosis: Biological and clinical perspectives. Expert. Opin. Med. Diagn..

[28] Moreno M.J., Ling B., Stanimirovic D.B. (2020). In vivo near-infrared fluorescent optical imaging for CNS drug discovery. Expert. Opin. Drug Discov..

[29] Cheng Z., Wu Y., Xiong Z., Gambhir S.S., Chen X. (2005). Near-infrared fluorescent RGD peptides for optical imaging of integrin alphavbeta3 expression in living mice. Bioconjug. Chem..

[30] Huang R., Vider J., Kovar J.L., Olive D.M., Mellinghoff I.K., Mayer-Kuckuk P., Kircher M.F., Blasberg R.G. (2012). Integrin alphavbeta3-targeted IRDye 800CW near-infrared imaging of glioblastoma. Clin. Cancer Res..

[31] De S., Razorenova O., McCabe N.P., O’Toole T., Qin J., Byzova T.V. (2005). VEGF-integrin interplay controls tumor growth and vascularization. Proc. Natl. Acad. Sci. USA.

[32] Schnell O., Krebs B., Wagner E., Romagna A., Beer A.J., Grau S.J., Thon N., Goetz C., Kretzschmar H.A., Tonn J.C. (2008). Expression of integrin alphavbeta3 in gliomas correlates with tumor grade and is not restricted to tumor vasculature. Brain Pathol..

[33] Cheng Z., Levi J., Xiong Z., Gheysens O., Keren S., Chen X., Gambhir S.S. (2006). Near-infrared fluorescent deoxyglucose analogue for tumor optical imaging in cell culture and living mice. Bioconjug. Chem..

[34] Ma Y., Lee Y., Best-Popescu C., Gao L. (2021). High-speed compressed-sensing fluorescence lifetime imaging microscopy of live cells. Proc. Natl. Acad. Sci. USA.

[35] Bower A.J., Li J., Chaney E.J., Marjanovic M., Spillman D.R., Boppart S.A. (2018). High-speed imaging of transient metabolic dynamics using two-photon fluorescence lifetime imaging microscopy. Optica.

